# Application of CRISPR Tools for Variant Interpretation and Disease Modeling in Inherited Retinal Dystrophies

**DOI:** 10.3390/genes11050473

**Published:** 2020-04-27

**Authors:** Carla Fuster-García, Belén García-Bohórquez, Ana Rodríguez-Muñoz, José M. Millán, Gema García-García

**Affiliations:** 1 Molecular and Cellular and Genomics Biomedicine Research Group, Instituto de Investigación Sanitaria La Fe (IIS La Fe), 46026 Valencia, Spain; c.fustergarcia@gmail.com (C.F.-G.); bg.bohorquez95@yahoo.es (B.G.-B.); rodriguezmunoz.ana@gmail.com (A.R.-M.); gegarcia@ciberer.es (G.G.-G.); 2 Rare Diseases Joint Unit, CIPF-IIS La Fe, 46026 Valencia, Spain; 3 Centre for Biomedical Research on Rare Diseases (CIBERER), 46026 Valencia, Spain

**Keywords:** retinal diseases, gene editing, CRISPR, cellular models, animal models, variants of unknown significance, functional studies, variant validation

## Abstract

Inherited retinal dystrophies are an assorted group of rare diseases that collectively account for the major cause of visual impairment of genetic origin worldwide. Besides clinically, these vision loss disorders present a high genetic and allelic heterogeneity. To date, over 250 genes have been associated to retinal dystrophies with reported causative variants of every nature (nonsense, missense, frameshift, splice-site, large rearrangements, and so forth). Except for a fistful of mutations, most of them are private and affect one or few families, making it a challenge to ratify the newly identified candidate genes or the pathogenicity of dubious variants in disease-associated loci. A recurrent option involves altering the gene in in vitro or in vivo systems to contrast the resulting phenotype and molecular imprint. To validate specific mutations, the process must rely on simulating the precise genetic change, which, until recently, proved to be a difficult endeavor. The rise of the CRISPR/Cas9 technology and its adaptation for genetic engineering now offers a resourceful suite of tools to alleviate the process of functional studies. Here we review the implementation of these RNA-programmable Cas9 nucleases in culture-based and animal models to elucidate the role of novel genes and variants in retinal dystrophies.

## 1. Introduction

Inherited Retinal Dystrophies (IRDs) comprise of a diverse group of vision loss diseases of genetic origin, generally due to the progressive death of the photoreceptors. Individually, these diseases have a low incidence among the population, yet together they present a prevalence of about 1 in 3000 [1].

The clinical classification of these disorders is based on the particular type of cells that are primarily affected (cones, rods, retinal pigment epithelium or inner retinal cells) giving rise to the distinctive clinical manifestation. Typical symptoms may include a reduction of the visual field (central or peripheral), visual acuity, color perception, night vision, or photophobia. However, the presentation of these clinical traits is characteristic of each specific disorder, even though some of the phenotypes display certain overlapping of the features [2,3,4]. Some of the most common IRDs are Retinitis Pigmentosa (RP), Stargardt Disease (STGD), Leber Congenital Amaurosis (LCA), and even syndromic forms such as Usher Syndrome (USH).

IRDs are genetically very heterogeneous since there are at least 271 genes currently associated to one or more of the diseases comprised in this group (according to RetNet, accessed March 2020). Moreover, several types of inheritance patterns can be found among this set of eye disorders, including autosomal recessive, autosomal dominant, X-linked, mitochondrial, and even some digenic forms have been proposed [5]. In addition, there is a wide mutational spectrum for these diseases, being most pathogenic variants private, with only a few exceptions having a higher representation, such as the p.Glu767Serfs*21 and p.Cys759Phe in *USH2A*; p.Gly1961Glu in *ABCA4*; p.Pro347Leu in *RHO*, or p.Cys998* (c.2991+1655A>G) in *CEP290* [6,7,8,9,10,11]. Furthermore, many of the genes (and mutations) associated with IRDs present inter- and intra-family phenotypic variability, with some genes even showing incomplete penetrance [12,13].

All these factors make both the clinical and genetic final diagnosis of IRDs cases challenging. To date, the gold-standard for the genetic characterization of patients is by means of high-throughput sequencing (HTS), usually through custom designs targeting known associated genes [14,15]. This method renders a considerable number of novel genetic variants whose clinical interpretation is initially assessed with in silico prediction software, segregation analysis, concurrence in other affected families and prevalence among the general population. However, often these mutations (particularly missense) remain officially classified as variants of unknown significance (VUS) or, at most, as possibly pathogenic. Therefore, on many occasions, functional assays are also expected to confirm the deleteriousness of a proposed variant, even more if a new candidate gene is involved. 

**Figure 1:**
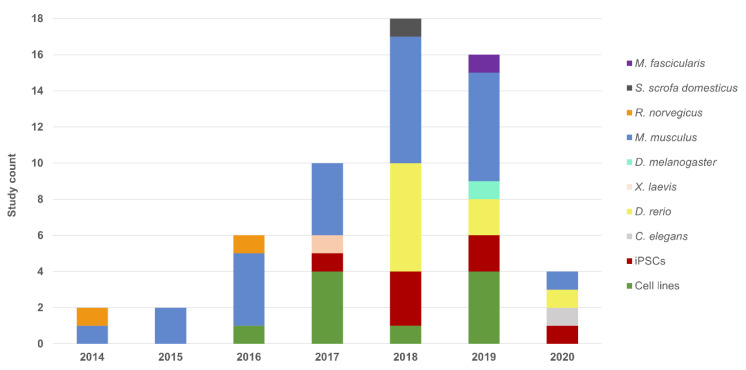
Chart of Inherited Retinal Dystrophies (IRD) related studies using the CRISPR technology for variant interpretation and disease modeling. A growing trend in the number of works that use this editing system is observed from the year 2014 to the first quarter of 2020, as well as a diversification in terms of the models used.

In this review, we address the CRISPR system as an asset for functional validation of VUS and for deepening into the pathogenesis role of known and novel IRD genes, given it is the increased interest in the field (Figure 1).

## 2. The CRISPR Toolkit—Initial Steps

The CRISPR system has overturned research on life sciences as a whole, given its broad applicability in many areas of biology. Even though the method represents a major breakthrough for potential therapeutic-aimed gene-editing techniques, it has also transformed the basic research field just by simplifying and speeding up the process of site-directed mutagenesis.

The CRISPR system (in its simplest form) relies on two main components, namely the Cas9 protein and a guide RNA (gRNA), which together form a complex that specifically targets a desired DNA locus [16]. This happens due to the hybridization of the complementary sequence of the gRNA that steers the Cas9 endonuclease towards the precise spot. 

Hence, the design of this sequence is pivotal for the edition outcome and it must adhere to certain guidelines. First, in order for the nuclease to be able to recognize and attach to the pertinent locus, a particular short nucleotide string must be adjacent to the gRNA-hybridization sequence in the host genome: the protospacer adjacent motif (PAM). The Cas9 from Streptococcus pyogenes (SpCas9) is the most commonly used and it requires a 3′ NGG motif. However, there are now many other nuclease versions either genetically engineered or coming from other organisms, such as Cpf1 or xCas9, that require different PAMs and feature other distances to the cleave point, thereby expanding the range of target possibilities [17,18,19]. Second, the choice of gRNA sequence, which should be 18–25 nt long, is determinant for the on-target efficiency and specificity, this latter translated as the potential of the fragment to mate with highly similar sequences throughout the genome (off-targets) producing unwanted DNA modifications. Fortunately, there is an increasing availability of computational tools that aid in the optimization of the design [20], as well as updated databases of these resources keeping pace with their rapid evolution [21].

Following site recognition, the nuclease cleaves the DNA and the resulting double-stranded break (DSB) is subsequently repaired mainly by two possible cellular mechanisms. The most prevalent pathway is the non-homologous end-joining repair (NHEJ), in which the cleaved ends are simply re-ligated. This is an error-prone mechanism that leaves small indels around the breaking point, due to some end resection of the strands and/or nucleotide additions [22]. Nonetheless, this somehow uncontrolled mutational outcome is of great profit when aiming for an easy and rapid generation of a knock-out or for gene disruption starting at specific points and indifferent to the following untranscribable sequence. Even more, the dual induction of DSBs using pairs of CRISPR complexes can readily replicate large structural variants, such as deletions, insertions, or translocations [23,24]. This might be the preferred strategy to confirm new candidate genes or to evaluate the specific role of its encoding protein in the molecular mechanisms of a certain cell or tissue.

The other method is known as the homology-directed repair (HDR), in which the damage is resolved through homologous recombination if a template is available; yet, even under these conditions, NHEJ still exceeds HDR. This is the method harnessed to generate precise nucleotide changes in the target DNA sequence by providing a carefully designed template, which can serve as means to either correct or introduce the underlying genetic defect of a disease. Most of the strategies used to knock-in make use of single-stranded oligonucleotides (ssODNs) or plasmids with long homologous arms to the region and the desired changes in the core as a repair template [25]. This precise method is the one usually employed to assess the impact of specific DNA changes presumed to be disease-causing.

Both the NHEJ and HDR pathways allow the directed modification of a specific DNA site while preserving the rest of the genome intact. Thus, the two CRISPR-induced strategies can be used to evaluate the pathogenicity of variants by enabling the fast generation of animal or cell/tissue-based models (as will be next discussed), spanning from days in the case of cell systems and simpler organisms like worms or flies, to weeks in vertebrate models and complex tissue-mimicking in vitro models. The strategies to address the distinct genes and mutations can vary significantly according to the appertaining form of IRDs (Table 1), just as the delivery methods differ depending on the type of targeted cells or organism (Table 2).

Regarding the latter issue, not all means are suitable for every target type. The nuclease/gRNA can be delivered via (i) plasmids or viral carriers, which first need to be imported to the nucleus for the complex to be transcribed; (ii) as mRNA molecules, cytosolically translated; (iii) or as a pre-assembled ribonucleoprotein (RNP) enabling immediate action. Except when virally packaged, these forms can also be released using different techniques, such as electroporation or direct injection for naked delivery, lipofection, or with other coating organic and inorganic nanoparticles. RNPs and mRNA molecules are preferable, since their transient expression reduces off-target activity [26,27,28], and also because of the harmful potential of vectors, given the intrinsic cytotoxicity of plasmids and the DNA integration of some viral vehicles [29,30]. In addition, the readiness of RNPs and the fact that this pre-built method has been proven to protect the gRNA from degradation render this option as the most efficient [31]. However, even though most cultured cells may take any of these procedures in, the more complex in vivo approaches are not that tolerant and mostly conducted via embryo microinjection or are viral-mediated [32,33].

## 3. Cellular Models

### 3.1. Cell Lines

Immortalized cell lines, due to their ease of handling, are a valuable tool to study the function of selected genes in vitro and to explore the impact of certain mutations (Table 1).

The origin of some broadly used cell lines, such as HEK293 or HeLa, is very different from cells implicated in IRDs but they can be equally used to study the function of some genes involved in these blinding disorders [84,85,86,87]. Among others, HEK293 cells have been used by Fuster and collaborators to assess the efficiency of diverse RNA guides designed to target p.Glu767Serfs*21 and p.Cys759Phe mutations in *USH2A* by CRISPR/Cas9 before using them in patient-derived fibroblasts, widely known to be more difficult to manipulate and transfect [36]. A similar strategy is applied when the main goal is to study the validity of the CRISPR system for gene editing in a mouse model. As a first step, usually, researchers test the designed RNA guides in manageable culture cells of mouse origin, such as the Mouse Neuro 2A (N2A) cells, derived from a mouse neuroblastoma, or mouse embryonic fibroblasts [40,41].

Other cell lines seem to be more appropriate as models for the study of pathophysiological mechanisms of IRDs, since they have a similar origin to the retinal tissue affected in these diseases. The immortalized mouse cone photoreceptor-derived 661W cell line has been widely used as a model to study the processes involved in the retinal degeneration, such as oxidative stress and cell death [88,89,90,91,92]. The 661W cell line has been used by Ji-Neng and colleagues to decipher the molecular pathways associated to *RP9* gene mutations, a pre-mRNA splicing factor responsible for autosomal dominant RP (adRP) [37]. By using the CRISPR/Cas9 system, these authors generated a *RP9* knock-out and a knock-in model for the p.His137Leu human mutation, which allowed them to demonstrate that mutations in the *RP9* gene lead to a reduction in cell proliferation and migration as well as a dysregulation in the expression of some downstream regulated genes [37].

Another common cell line derived from the female retinal pigment epithelium (RPE), hTERT-RPE1, has been used for the study of the molecular mechanisms involved in IRDs and other ciliopathies due to their origin, the presence of cilia, and the capacity to reciliate under certain conditions [93]. This cell line was chosen to investigate the role of *RP2*, a gene causative of X-linked retinal degeneration, because of its motility in culture. The CRISPR/Cas9 *RP2* knock-out model showed a reduced motility compared to *RP2* wild type cells [38]. hTERT-RPE1 was also utilized to study how mutations in *RPGR*, another gene causing X-linked RP, lead to photoreceptor loss [39]. To achieve this aim, knock-out cell lines for *RPGR* and several interactor genes (*PDE6D*, *INPP5E* and *RPGRIP1L*, all three are also involved in retinal degeneration) were generated with the CRISPR/Cas9 system allowing to demonstrate that PDE6D is necessary for a correct ciliary localization of prenylated proteins such as RPGR and INPP5E, but that RPGR is not essential for the localization of INPP5E in the cilia [39].

Primary cell lines harvested from patients or healthy donors represent a valid alternative to common cell lines for the study of retinal degeneration. It is important to note that these are usually difficult to handle, given their limited growth in culture conditions and reduced efficiency of transfection. However, primary cells enable the preservation of the genetic background of source patient, and fibroblasts are largely used in this sense. Fuster and collaborators were able to edit by CRISPR/Cas9 the most prevalent mutation in the *USH2A* gene, p.Glu767Serfs*21, in fibroblasts obtained from a patient homozygous for the mutation [36]. 

Recently, Yang et al., used a pool of primary human keratinocytes to study the effect of mutations in the *EXOSC2*, a gene encoding for one cap protein in the RNA exosome (RRP4) and associated to SHRF syndrome (short stature, hearing loss, retinitis pigmentosa and distinctives facies), which is a novel syndromic form of IRDs [42,94]. By generating an *EXOSC2* knock-out model, the authors demonstrated that keratinocytes have a reduced proliferation that might explain early skin aging observed in patients with this syndrome. 

### 3.2. Induced Pluripotent Stem Cells

Ever since the arrival of induced pluripotent stem cells (iPSCs) [95], biomedical research has taken a big leap, especially in regard to therapeutic and disease model applications. This technology allows the reprogramming of virtually any somatic human cell population to a complete pluripotent state, with the potential to subsequently generate tissue-specific progenitor cells or even a completely differentiated line [96]. This poses a great resource to explore the molecular defects of a certain pathology, including those related to differentiation mechanisms. Studies within this cellular scope consist of side-by-side assays using diseased and normal sets [97]. Rationally, those would require multiple unrelated samples to normalize the individual genetic variability of each lineage and the one resulting from the dedifferentiation process itself. 

Yet to study the repercussion of a particular variant, this proceeding would turn out to be unavailing for two main reasons. First, with respect to IRDs, the low reoccurrence of disease-causing mutations would frustrate a significant statistical inference due to the poor number of independent samples in the analysis. Second, even if an adequate stock could be obtained, the prospect of any other linked VUS as the actual disease trigger would still call the results into question [98]. Indeed, even in iPSCs from the same donor, phenotypic differences have been detected and they are amplified if derived from unrelated individuals [99], encouraging the search for methods to keep this variability at a minimum.

In this matter and as previously commented, the use of CRISPR-based genome editing permits the introduction of precise genetic changes in these iPSCs without altering the rest of their genomic sequence, thereby providing isogenic cell line pairs only differing in the mutation of interest. This has a great relevance for the assessment of variant pathogenicity, since it enables the comparison of two cell populations with identical genetic backgrounds and, thus, the impact appraisal of a specific single DNA lesion in the cellular phenotype [100]. 

To do so, there are two essential routes [98]. The first strategy would be using patient-derived cells to repair the putative disease-causing mutation for the later monitoring of the corrected and unaltered line. However, this might be rather treacherous in some circumstances, since any detected differences would only prove the involvement of the variant yet not rule out the contribution of other changes throughout the genome. This presumption, though, might only apply for other more complex diseases in which oligogenic inheritance patterns are not uncommon, unlike with IRDs, which usually are explained by mutations in single genes. Even so, the influence of possible modifiers should not be disregarded, as evidenced by the incomplete penetrance and clinical variability of some alleles in certain retinal disorders [101,102,103,104]. Certainly, a comparative of isogenic lines should likewise serve to prove some presumed digenic or gene-modifying scenarios by testing mutation combinations. The second approach consists of the reverse procedure, in which cells obtained from healthy individuals are genetically edited to harbor the variant of study to determine if it is sufficient to produce the pathogenic phenotype regardless of the rest of genome variation.

In either case, genotype-specific disease modeling in cell cultures is much easier for dominant disorders, where only one of the two alleles need to be edited. It should be noted that the positive activity of the CRISPR complexes within a cell does not ensure their operation on both chromosomes, being the chances of producing a single vs. a dual modification greater. Likewise, the introduction of a homozygous variant under a dominant premise could bias the experiment conclusions by producing a stronger phenotype. Thus, in any way, a clone selection step is required to obtain a homogeneous cell population, even though the process might be more swift when aiming for dominant traits.

It should be recalled, that the CRISPR technique does have secondary effects in the form of potential off-targets when the designed gRNA presents a highly similar sequence to other loci of the genome, in which the complex could act by producing DSBs and, hence, further genome modifications [105]. The occurrence of these by-products is neither fixed nor abundant, yet any of these unintended genetic alterations could give rise to the anticipated differing phenotype, misattributed to the intended predesigned change. Thus, a full post-edition genome survey should be mandatory to confidently dismiss these potential changes as the actual pathogenic cause.

In order to deem a variant as a disease causative, or at least contributing, some sort of ponderable molecular or cellular trait differing from the control cell line is necessary. A good example for the use of these CRISPR editing techniques is the recent work conducted by Sanjurjo-Soriano and colleagues, where they used patient-derived iPSCs carrying the two *USH2A* prevalent mutations (p.Glu767Serfs*21 and p.Cys759Phe) and the corrected isogenic counterpart, and detected abnormal mRNA levels associated to the variants (see other studies in Table 1) [49].

### 3.3. Retinal Organoids

Despite iPSCs being such an evident profitable and manageable trial source, the method still falls short of typifying the more complex cellular networks of whole organs. Therefore, the scrutiny of merely the ultimately affected cell type might not prove to be conclusive, as the pathological mechanisms of a disease are not always attributable to its own morphology or transcriptome, but also to physiological and molecular interactions with surrounding cells. Given that the retina consists of a sophisticated stratum of neuronal cells, this is probably the case for the study of some variants in genes responsible for IRDs.

An alternative, yet more complicated, culture-based strategy to overcome this issue is the use of the recently emerged retinal organoids (ROs). These optic cups consist of the three-dimensional disposition of all retinal cells that reproduces the spatial arrangement and the development of the tissue [106]. Consequently, this rudimentary organ-like structure offers a more realistic model to study the impact of variants on a larger scale, yet still under in vitro conditions. In addition, as the organoid formation mimics the natural time-framed differentiation of the eye neuronal layers, it allows for the investigation of the consequences of a DNA change in the retinogenesis [107]. Moreover, the use of ROs might even add other read-out options for the functional appraisal, since photoreceptors in these models seem to analogously respond to light stimulus and to recapitulate the signal transmission towards the inner retinal cells [108,109].

ROs by themselves have major limitations, since they actually resemble an embryonic-staged retina. Cells are not fully developed, as evidenced in the incomplete formation of the outer and inner segments of the photoreceptors, and the 3D structure lacks vascularization and other cell types that are present in the native organ, like microglia and the RPE [110]. However, it would seem that a recently described approach is able to circumvent these shortcomings and more accurately recapitulate the in vivo scenario by co-culturing RPE sheets and ROs within a controlled microperfusion system [111]. Labelled as retina-on-a-chip (RoC), it promotes maturation of the photoreceptors by allowing the interaction of their pseudo-outer segments with the RPE and due to the constant flux of nutrients provided by the pumping platform. Hence, this evolved scaffold-like model now stands as a promising prototype recapitulating the in vivo architecture of the retina to study the inherent cellular mechanisms, effects of genome manipulations, drug treatments, and so forth.

Alike with the iPSCs, the blend of the CRISPR tools with the availability to obtain ROs would boost the cogency to validate candidate VUS. Comparatives of patient and control-derived ROs can present enough evidence supporting the deleteriousness of certain mutations [112]. However, there is also proof that 3D cups stemming from different cell lines display variation on their development [113], possibly determining their later stability and electrophysiological properties. Hence, the use of isogenic ROs should be encouraged to remove all uncontrolled variability that may bias the variant pathogenicity determination, something that can be attained using CRISPR-pre-processed iPSCs before their differentiation into optic cups.

The study of Buskin et al. is one of several examples implementing these programable nucleases in such optic cups (Table 1) [44]. The researchers produced several ROs from patient-derived cells with mutations in the *PRPF31* gene, responsible for adRP. In parallel, they corrected these genetic changes in iPSCs prior to RO differentiation, providing a peer 3D-source for the appraisal of any molecular differences imputable to the variants. Indeed, results not only allowed the confirmation of *PRFP31* involvement in ciliogenesis regulation, but also a consistent molecular characterization of the pathogenesis.

All these properties place the ROs as the more reliable ‘disease-in-a-dish’ model to date, and it might be more than enough to ratify the pathogenicity of a suspected mutation. Nonetheless, since these primitive organs do not engage in further physiological processes and anatomic elements of the organism, they still are lacking in replicating the whole disease picture.

## 4. Animal Models

### 4.1. Caenorhabditis elegans

*Caenorhabditis elegans* (*C. elegans*) is a nematode that plays an important role in biological research. Back in 1965, the Nobel prize-laureated Sydney Brenner chose this worm as a model to study animal development and behavior, and it has ever since been considered an excellent model to study biological processes due to several advantages: its rapid life cycle, its small size, the ease in terms of laboratory management, its transparency (which facilitates the use of microscopy in vivo), and the fact that despite its simplicity, it is still a complex multicellular organism with a variety of tissues and organs. In sum, *C. elegans* is a model organism that brings together the advantages of in vitro and in vivo research.

To date, mutations in 22 genes have been related with adRP (RetNet, accessed March 2020), and seven of these genes encode splicing factors. These seven genes are *PRPF3*, *PRPF4*, *PRPF6*, *PRPF8*, *PRPF31*, *SNRNP200* and *RP9*. All of them, except for *RP9*, encode highly conserved proteins between *C. elegans* and humans [114]. Photoreceptor cells are characterized by an intense transcriptional activity and metabolic rate [115] and, even though they are absent in *C. elegans*, the animal does present other cells that have similar increased metabolic rate and high transcriptional levels during the larval phase [116,117]. 

The clinical variability that characterizes IRDs can be explained by the existence of genetic modifiers [118]. In this context, Kukhtar et al. generated mutant strains to mimic two pathogenic mutations reported in PRPF8 and SNRNP200 by CRISPR/Cas9 to identify potential adRP modifiers and to explore therapies that may slow the disease progression [51].

### 4.2. Drosophila melanogaster

*Drosophila* has good genetic tractability and 65% of the human genes responsible for a disease has a homolog in this fly model, including most of the genes involved in IRDs [119]. In 1995, *Drosophila* was used for the first time as a model to decipher the mechanisms by which mutations in *RHO* caused retinal degeneration, demonstrating the great potential of this model for this group of diseases [120]. Recently, Yang and collaborators generated a knock-out of *EXOSC2* in the fly model assisted by the CRISPR tools and showed that some patterns, such as the autophagy, were altered in SHRF syndrome due to mutations in this gene (Table 1) [42].

### 4.3. Xenopus

*Xenopus* is an amphibian broadly used as a model to study development and cell biology because of its well-preserved development and genetic proximity to higher vertebrates. Further characteristics such as the size, external development of the embryos, type of breed, and the large number of progeny, make *Xenopus* a suitable animal model [121,122]. There are two main species that are used in research, namely *X. laevis* (allotetraploid genome) and *X. tropicalis* (diploid genome) [122,123], and it is the former, the one that has been used to model retinal dystrophies, with a special focus on the rhodopsin gene. Knock-out models have been generated by the microinjection of Cas9 mRNA and sgRNAs into single cell embryos [83]. Likewise, three knock-in models were produced in the same study using an external template donor. Despite the scarce research on *Xenopus* oriented to IRDs, the available results demonstrate that the organism can be genetically manipulated using the CRISPR/Cas9 system and be used to model eye diseases (Table 1).

### 4.4. Zebrafish

Another animal model widely used in research on IRDs is the zebrafish (*D. rerio*), a freshwater fish belonging to the family of *Cyprinidae.* This vertebrate model organism has been used for many years in developmental studies, toxicology, preclinical drug development, or molecular genetics, due to several beneficial characteristics like its fast life cycle, external larvae development, small size, transparency and easy maintenance and breeding [124,125,126,127,128]. However, this model has most of its genome duplicated [129], which complicates the editing of all functional gene copies. In addition, at least 70% of the human disease-related genes have their ortholog in zebrafish, which eases the study of their functions and involved molecular mechanisms, assuming an equal role in both species [130]. Moreover, compared to other vertebrate models commonly used, zebrafish has a higher number of offspring, which supposes an additional advantage [131,132].

Besides these general positive traits, it should also be noted that this organism is able to regenerate its retinal layers and some neural subtypes, thereby seeming to be a good research model for retinal diseases [133,134,135,136,137,138,139].

Additionally, following its whole genome sequencing [130], reverse genetic and other molecular tools have thrived, making it possible to perform, for example, retroviral-mediated insertional mutagenesis or CRISPR/Cas9 technology [140,141,142]. Hence, the emergence of new editing techniques has expanded the range of possibilities in the field of candidate gene or variant assessment. In fact, the zebrafish was the first model used to demonstrate the in vivo feasibility of the CRISPR/Cas9 system for genome editing, achieving up to 50% of on-target edition rate [143].

Moreover, this organism is a widely used model for functional assays in IRDs, usually based on the development of gene knock-downs or knock-outs [52,53,58], some lately generated by means of the genomic editing system here reviewed (Table 1). As an example, Minegishi and colleagues decided to confirm the involvement of the *CCT2* gene in LCA [78], suspected due to identification of the compound heterozygous mutations p.Thr400Pro and p.Arg513Hisby whole exome sequencing (WES) in the affected members of a sole Chinese consanguineous family [55]. The produced cct1-L394-7del line by CRISPR/Cas9 demonstrated that the mutated *cct2* gene implied serious disabilities in zebrafish, highlighting its important role in this vertebrate model. Furthermore, the homozygous cct2-L394H-7del mutant showed a phenotype rescue when injected with human wild type *CCT2*-coding RNA. In conclusion, this study served as a candidate gene validation, showing that mutations in the *CCT2* ortholog result in a phenotype that resembles the one in human LCA patients.

A similar procedure was performed by Van De Weghe et al. in 2017 [144]. A large cohort of patients diagnosed with Joubert Syndrome (JS), were studied by WES, which led to the detection of different variants in *ARMC9*, including point mutations and whole exon deletions. Initial assays revealed that the gene was highly expressed in the ciliary basal body of ciliated cells, as alike with other genes involved in cilium function that are also upregulated. Leveraging the presence of an *ARMC9* ortholog in zebrafish with at least 72% protein similarity, the authors introduced frameshift mutations in *armc9* by the CRISPR/Cas9 technology to investigate the resulting phenotypes. Small pairs of gRNAs were co-injected to target exons 4 and 14–15 generating several mutant zebrafish strains. The resulting phenotypes confirmed that mutations in the *ARMC9* gene are responsible for ciliopathy phenotypes in JS patients. 

Knock-in studies to evaluate the specific IRD-causing variants have also been conducted in zebrafish. For instance, Dona and colleagues were able to reproduce two mutations responsible for USH in *USH2A* (p.Cys780Glnfs*32 andp.Ala5174fs*), providing finer strains modeling the disease to analyze the pathogenic molecular mechanisms underlying RP [56].

In a different study, another mutation in the same gene was introduced for two purposes, the pathogenic validation of the variant in this aquatic model and its later use for a therapeutic approach [57]. In humans, the deep intronic p.Lys2532Thrfs*56 mutation (c.7595-2144A>G) creates a potential donor splice-site in intron 40 leading to the introduction of a pseudoexon in the mRNA [145], yet Slijkerman et al. concluded that this effect was not recapitulated in zebrafish, disclosing that splice-site recognition pathways are different in the human and zebrafish organisms [57].

### 4.5. Rodents

Regardless of the mutant generation procedure, the mouse (*Mus musculus*) is by far the most commonly used organism for disease modeling. This also applies to human eye disorders, given that the mouse and human eye share many anatomic and physiologic characteristics [146]. Besides the possibility to carry out reproducible developmental and invasive studies, mice also allow relatively easy ophthalmological characterizations of the diseases through different tools, such as indirect ophthalmoscopy, fluorescein angiography, optical coherence tomography, or electroretinography. In addition, the mouse has a rather short lifespan considering its condition of mammalian organism, which is convenient in terms of model generation and later monitoring of the natural progression of the disease of study.

Despite all the progress made in the last years, the delivery of the CRISPR components remains a challenge, and mice pose a good model to investigate later human-applicable possibilities. To date, adeno-associated virus (AAV) vectors are the most effective method for delivering gene therapy compounds to retinal cells [147,148]. Following a knockdown strategy with easily detectable outcomes, Hung et al. tested the viability to target retinal cells in vivo through intravitreal administration of CRISPR AAV2 vectors inThy1-YFP mice, a reporter strain expressing a fluorescent protein in the ganglion, amacrine and bipolar cells [66]. 

A different study directed to photoreceptors employed a dual-AAV2/8 system for the delivery of CRISPR components to disrupt *Nrl*, a gene dictating rod development during development, in three different RP mice models (*rd10*, *Rho* KO and *Rho* P347S) [75]. Yu and colleagues established that *Nrl* ablation caused rods to acquire cone characteristics, mitigating the degeneration process by extending the survival of these converted-rods and preserving cone function in those cases where rod-specific mutations are accountable for the dystrophy. Other studies using this viral delivery method have gone beyond and added to the design a self-inactivating trait of the CRISPR system based on the autotargeting of the Cas9 coding sequence in the cassette once expressed, in order to limit the expression in the retina of the nuclease and, thereby, reducing undesirable off-target events, potential toxicity and immune response against Cas9 [80,149]. Another in vivo Cas9-restriction method, once again aimed to regulate the *Nrl* gene, was presented by Moreno et al., who devised a doxycycline-inducible nuclease expression construct [76].

CRISPR/Cas9-mediated NHEJ repair has quickly become a routine method for creating gene knock-outs in animal models, as it is a considerably simpler and more productive approach than the HDR-based knock-in process. Accordingly, many authors have demonstrated that generating knock-out mouse models by the CRISPR/Cas9 editing system is an efficient method to determine the pathogenic role of new IRD-causing candidate genes and to characterize the disease mechanisms associated with their mutations in them [62,69,70,74,78]. As an example, a recently identified homozygous missense variant (p.Trh58Met) in *HKDC1* in two unrelated RP families by WES postulated the gene as candidate for the disease, and the disruption of *Hkdc1* in mice using these programable nucleases resulted in a reduced scotopic electroretinogram response and thinner outer nuclear layer similar to the human patients [71]. Hence, these findings enabled the final linking of the gene to arRP.

For diseases caused by a gain of function, dominant negative variants or increased gene copy numbers, CRISPR/Cas9 can be used to selectively suppress the mutated allele that produces toxicity in the retina. In this line, an interesting proceeding was conducted in a rat model of adRP caused by the p.Ser334*stop-gain mutation in *Rho*, which was tackled by introducing an allele-specific gRNA/Cas9 complex by means of retina electroporation of new-born animals, preventing retinal degeneration [82].

Genome-editing nucleases can also be used to deal with regulatory elements. Seruggia et al. carried out a successful inactivation of a non-coding regulatory locus upstream the *Tyr* gene, employed by microinjecting mouse fertilized eggs with a combination of Cas9 and two sgRNAs targeting unique flanking sequences of the region [65]. Wang et al. also used two-sided NHEJ-mediated DSBs to produce a fundamental 108bp deletion in a *cis*-regulatory element controlling the *Blimp1* expression, whose encoded protein is in itself a transcription factor critical for the rod versus bipolar cell differentiation, abolishing the function of the gene [61].

It must be considered that sometimes null homozygous alleles that in humans derive from autosomal recessive phenotypes can be lethal to the mouse, consequently hampering the disease emulation. This happened to Zhong and colleagues, who used the CRISPR/Cas9 system to generate mouse *Kcnj13* null alleles to verify the pathogenic role of the human ortholog *KCNJ13*, a gene that was associated with LCA [64]. The study exposed the fatality of the complete loss of the gene, yet the retinal degeneration in animals withholding a portion of cells with the preserved wild type gene could still be used to confirm the requirement of *KCNJ13* for photoreceptor survival. Moreover, these results revealed the potential use of mosaicism for in vivo gene functional validation in otherwise lethal mutations in mice.

Knock-in approaches using the CRISPR/Cas9 technology are carried out in this animal too, alike with other models, either to create more genuine disease-models or to interrogate the effect of specific genetic variants. Arno et al. exemplified this application by replicating in mice the p.Leu135Pro mutation in *Reep6* in homozygosis via HDR following CRISPR/Cas9-mediated DSB, which reproduced the RP phenotype and, therefore, corroborated human *REEP6* implication in the disease [68].

Almost all pathogenic mutations described in *RPE65* are recessively inherited but the p.Asp477Gly variant has been reported to cause adRP in families of Irish heritage [150,151]. To prove the dominant impact of this particular mutation, Li and colleagues generated the mutation knock-in model in the mouse, again employing CRISPR/Cas9 resources [63]. They observed that heterozygous mice did not show any anomalous visual function, yet under homozygous conditions their retina did display signs of degeneration when subjected to light stress. The absence of an aberrant phenotype does not refute the dominance of the genetic change, given the similar outcome for other variants patently responsible for adRP, even though the presence genetic disease-modifiers could have been in fact neglected in the genetic screening of the patients with the p.Asp477Glymutation. Therefore, this case actually exposes the CRISPR knocking-in as a valid method to confirm but not discard proposed pathogenic effects of mutations.

### 4.6. Pig

Domestic pigs (*Sus scrofa domesticus)* share several key similarities with humans in terms of their body size, anatomy, physiology, genetics, pathophysiological responses, and diet [152]. Its use in biomedical research has some advantages like their favorable breeding characteristics. Pigs mature relatively quickly for such a large species, have a short gestational period, and produce extensive litters [153]. In general, due to the cone-rich nature of the porcine retina, the pig is considered to be an ideal model for studying IRDs, especially cone-dystrophies [146]. Burnight et al. chose a transgenic pig model that carries the human p.Pro23His mutation to perform an AAV-mediated allele-specific CRISPR correction in vivo, delivering these viral constructs by subretinal injection [43]. 

### 4.7. Macaque

Nonhuman primates have a crucial role in translational studies due to their high similarity with humans in several aspects, such as genetics and physiology. Usually, this model is used in the last preclinical phase and before starting with clinical trials in humans [154]. This step is not always necessary given the accessibility to the other model organisms explained above, which can provide enough safety and efficiency evidence for some explored treatments. Among the genus, *Macaca fascicularis* has been used to analyze the viability of CRISPR/Cas9 design as a therapeutic approach to treat a type of autosomal dominant cone-rod dystrophy (CORD6) due to mutations in *GUCY2D*, an approach in mice [79]. Subretinal injections of AAV containing the CRISPR complex sequences have been used as a delivery method to specifically knock out the *GUCY2D* gene in macaque photoreceptors (Table 1). The tissue-restricted model was thoroughly characterized and the results obtained served the authors as proof of concept for a potential therapeutic application of these editing tools for other retinal diseases [79].

## 5. Conclusions

The key contribution of the CRISPR/Cas9 technology towards variant interpretation always falls on the relatively easy and quick reproducibility of mutations in an in vitro or animal model, allowing discernment of pathogenicity if any aberrant molecular or morphological changes result from its sole conception. 

This revolutionary editing technology is being increasingly used in genetics studies, keeping pace with updates of the technique that show a clear advance in its precision. This will lead to not only an improvement in the diagnosis ratio but also lead to the development of potential therapeutic options.

Taking advantage of the easy accessibility to the retina, research on non-viral delivery procedures should be fostered, aiming for hit-and-run alternatives that proffer more restrained outcomes in regard to off-target activity. This will be relevant to obtaining safer editing trials, not only with the sights set on therapeutic goals but also in terms of producing more robust disease-models and casting no doubts on their ex situ genetic integrity that, if compromised, could mask an expected phenotype. 

## Figures and Tables

**Table 1:** Studies using the CRISPR technology for functional validation of variants of disease modeling.

Model	Phenotype	Gene	Genomic Target	Aim	Delivery Method	Nuclease	Reference
^H^ HeLa	adRP	*RHO*	p.Pro23His	KO	Lipofection (plasmid)	SpCas9	[34]
^H^ HEK293FT	LCA	*CEP290*	p.Cys998*	KI	Lipofection (plasmid)	SpCas9	[35]
^H^ HEK293	USH2/arRP	*USH2A*	p.Glu767Serfs*21	KI	Lipofection (plasmid)	SpCas9	[36]
			p.Cys759Phe				
^M^ 661W	adRP	*Rp9*	Exon 5	KO	Fugene HD (plasmid)	SpCas9	[37]
			p.His137Leu	KI			
^H^ hTERT-RPE1	xlRP	*RP2*	Exon 2	KO	Fugene HD (plasmid)	nCas9 pairs	[38]
		*RPGR*	Exons 2 and 4	KO	Undetermined	uCas9	[39]
	sarRP	*PDE6D*	Exon 2				
		*INPP5E*	Exon 1				
	arCORD	*RPGRIP1L*	Exon 3				
^M^ NSC	arRP	*Pde6b*	p.Arg560Cys	^C^ KI	Nucleofection (plasmid)	SpCas9	[40]
*rd12* MEFs	LCA	*Rpe65*	p.Arg44*	^C^ KI	Electroporation (RNPs)	SpCas9	[41]
PD-Fibroblasts	USH2/arRP	*USH2A*	p.Glu767Serfs*21	^C^ KI	Nucleofection (RNPs)	SpCas9	[36]
PD-Keratinocytes	SHRF	*EXOSC2*	Exon 1 and 4	KO	Lentiviral transduction	SpCas9	[42]
PD-iPSCs	arRP	*MAK*	c.1513ins353	^C^ KI	Nucleofection (plasmid)	SpCas9	[43]
	LCA	*CEP290*	p.Cys998*	KO			
				KO	Electroporation (plasmid)	SaCas9	
				^C^ KI			
	adRP	*RHO*	p.Pro23His	KO			
				^C^ KI	Undetermined (plasmid)	SpCas9	
		*PRPF31*	p.Arg372Glnfs*99	^C^ KI	Lipofection (plasmid)	SpCas9	[44]
			Exon 7	KO	Nucleofection (plasmid)	SpCas9	[45]
		*PRPF8*	p.Pro2301Ser	^C^ KI	Electroporation (gRNA-plasmid and Cas9 mRNA)	Cas9-Gem	[46]
	xlRP	*RPGR*	p.His562Argfs*20	KI	Electroporation (plasmid)	SpCas9	[47]
	ESCS	*NR2E3*	p.Val41Alafs*23	KI	Lipofection (plasmid)	SpCas9	[48]
			p.Arg73Ser	KI	Electroporation (plasmid)		
	USH2/arRP	*USH2A*	p.Glu767Serfs*21 p.Cys759Phe	KI	Nucleofection (plasmid)	eSpCas9	[49]
	XLRS	*RS1*	p.Arg209Cys	KI	Nanodiamonds (linear DNA)	SpCas9	[50]
*Caenorhabditis elegans* (nematode)	adRP	*prp-8*	p.Arg2310Gly	KI	Injection (RNPs)	SpCas9	[51]
			p.His2309del				
		*snrp-200*	p.Val683Leu				
			p.Ser1087Leu				
*Danio rerio* (zebrafish)	arRP	*eys*	p.Gly1163Valfs*14	KO	Embryo injection (RNPs)	SpCas9	[52]
		*pcare*	p.Gly8Glu*19	KO	Embryo injection (RNPs)	SpCas9	[53]
	adRP	*rho*	p.Cys322Argfs*116	KO	Embryo injection (Cas9 mRNA and gRNAs)	SpCas9	[54]
	LCA	*cct2*	p.Leu394His-7del	KO	Embryo injection (RNPs)	SpCas9	[55]
	USH2/arRP	*ush2a*	p.Cys780Glnfs*32	KO	Embryo injection (Cas9 mRNA and gRNAs)	uCas9	[56]
			p.Ala5174*				
			p.Lys2532Thrfs*56	KI	Embryo injection (RNPs)	SpCas9	[57]
	ESCS	*nr2e3*	p.Leu162Glnfs*30	KO	Embryo injection (Cas9 mRNA and gRNAs)	uCas9	[58]
	arCD	*cacna2d4*	Undetermined	KO	Embryo injection (RNPs)	SpCas9	[59]
	adFEVR	*znf408*	p.His455Tyr	KI	Embryo injection (RNPs)	uCas9	[60]
*Mus musculus* (mouse)	Undetermined	*Blimp1*	B108 *cis*-regulatory module	KO	Electroporation—subretinal injection (plasmid)	SpCas9	[61]
	LCA	*Cep290*	p.Cys998*	KO	AAV transduction (subretinal injection)	SpCas9	[35]
			Exon 3	KO	AAV transduction (subretinal injection)	SpCas9	[62]
		*Rpe65*	p.Asp477Gly	KI	Embryo injection (Cas9 mRNA and gRNAs)	SpCas9	[63]
			p.Arg44*	^C^ KI	AAV transduction (subretinal injection)	SpCas9	[41]
		*Kcnj13*	Exon 2	KO	Zygote injection (Cas9 mRNA and gRNAs)	SpCas9	[64]
	OCA1	*Tyr*	5’ region	KO	Zygote injection (Cas9 mRNA and gRNAs)	SpCas9	[65]
	Thy1-YFP	*YFP*	5’ region	KO	AAV transduction (intravitreal injection)	SpCas9	[66]
	arRP	*Pde6b*	p.Arg560Cys	KI	Electroporation—subretinal injection (plasmid)	SpCas9	[40]
			p.Tyr347Ter	^C^ KI	Embryo injection (gRNA-plasmid and Cas9 protein)	SpCas9	[67]
		*Reep6*	p.Leu135Pro	KI	Embryo injection (Cas9 mRNA and gRNAs)	SpCas9	[68]
			Exon 4	KO	Embryo injection (Cas9 mRNA and gRNAs)	SpCas9	[69]
		*Arl2bp*	Exon 2	KO	Embryo injection (Cas9 mRNA and gRNAs)	SpCas9	[70]
		*Hkdc1*	Exon 2	KO	Undetermined (plasmid)	uCas9	[71]
	adRP	*RHO*	p.Pro23His	KO	Electroporation (plasmid)	SpCas9	[34]
					Electroporation (plasmid) and AAV transduction (intravitreal injection)	SaCas9 and SaCas9-KKH	[72]
		*Rho*/*RHO*	Exon 1	KO	AAV transduction (subretinal injection)	SpCas9	[73]
	arRP/sarRP	*Cwc27*	p.Lys338Glyfs*25	KO	Embryo injection (Cas9 mRNA and gRNAs)	SpCas9	[74]
	arRP/adRP	*Nrl*	Undetermined	KO	AAV transduction (subretinal injection)	SpCas9	[75]
							[76]
	RD	*Slc9a8*	Promoter	KO	AAV transduction (subretinal injection)	nmCas9	[77]
		*Usp45*	Exon 14	KO	Embryo injection (Cas9 mRNA and gRNAs)	uCas9	[78]
	adCORD	*Gucy2e*	Exon 2 and 4	KO	AAV transduction (subretinal injection)	SaCas9	[79]
	Thy1-YFP	*YFP*	Undetermined	KO	AAV transduction (subretinal injection	SpCas9	[80]
	XLRS	*Rs1*	p.Arg209Cys	KI	Nanodiamonds—intravitreal injection (linear DNA)	SpCas9	[50]
*Rattus norvegicus* (rat)	OCA1	*Tyr*	Exon 2	KO	Embryo injection (Cas9 mRNA and gRNAs)	uCas9	[81]
			p.Arg299His	KI			
	adRP	*Rho*	p.Ser334Ter	KO	Electroporation—subretinal injection (plasmid)	SpCas9	[82]
*Xenopus laevis* (frog)	adRP	*rho*	Exon1	KO	Embryo injection (Cas9 mRNA and gRNAs)	SpCas9	[83]
			Exon 5	KI			
*Drosophila melanogaster* (fly)	SHRF	*rrp4*	Exon 1 and 4	KO	Embryo injection (gRNA-plasmid into Cas9-expressing strain)	SpCas9	[42]
*Sus scrofa domesticus* (pig)	adRP	*RHO*	p.Pro23His	KO	AAV transduction (subretinal injection)	SaCas9	[43]
*Macaca fascicularis* (macaque)	adCORD	*GUCY2D*	Exon 2 and 4	KO	AAV transduction (subretinal injection)	saCas9	[79]

KO: Knock-Out; KI: Knock-In; MEFs: Mouse Embryonic Fibroblasts; HeLa: Henrietta Lacks Cell Line (Uterine Cell Variety); HEK293: Human Embryonic Kidney 293 Cells; HEK293FT: Fast Growing HEK293 Line Variant; 66W: Immortalized Cone Photoreceptor Cell Line; hTERT-RPE1: Immortalized Retinal Pigment Epithelial Cell Line; NSC: Primary Cultures of Neural Stem Cells; eSpCas9: Enhanced Specificity SpCas9; nCas9: Cas9 Nickase; SpCas9: Cas9 (Streptococcus pyogenes); uCas9: Undetermined Cas9; SaCas9: Cas9 (Staphylococcus aureus); SpCas9-Gem: SpCas9 Fused to Human Geminin Protein; SaCas9-KKH: SaCas9 Recognizing NNNRRT PAMs; nmCas9: Cas9 (Neisseria meningitidis); AAV: Adeno-Associated Virus; PD: Patient-Derived; ad: Autosomal Dominant; ar: Autosomal Recessive; sar: Syndromic Autosomal Recessive; xl: X-Linked; RP: Retinitis Pigmentosa; RD: Retinal Degeneration; CD: Cone Dystrophy; USH2: Usher Syndrome Type 2; XLRS: X-Linked Juvenile Retinoschisis; SHRF: Short Stature, Hearing Loss, Retinitis Pigmentosa and Distinctive Facies Syndrome; FEVR: Familial Exudative Vitreoretinopathy; ESCS: Enhanced S-Cone Syndrome; CORD: Cone-Rod Dystrophy; OCA1: Oculocutaneous Albinism Type 1; LCA: Leber Congenital Amaurosis; Thy1-YFP: Transgenic Mice Expressing Yellow Fluorescent Protein; Symbols: ^H^ Human-origin cells; ^M^ Mouse-origin cells; ^C^ Correction-purpose.

**Table 2:** Pros and cons of the CRISPR/Cas9 delivery methods described in this review.

Method	Advantages	Disadvantages
Microinjection	Liberated right into the cell High efficacy	Time-consuming Technique expertise
Electroporation	Normalized open-access protocols High effectiveness with plasmids	In vitro and ex vivo cell restriction Cell cytotoxicity Not all cells are susceptible
Lipofection	Works in many cell types Easy manipulation Inexpensive Reduced off-targets	Exclusive for cell culture Lysosome degradation
Nanodiamonds	Highly efficient delivery High biocompatibility Water solubility Fully accessible surface Inexpensive	Genotoxicity High pressure and temperature for synthesis Tissue distribution problems
AAVs	Low immunogenicity and cytotoxicity Reduced off-targets High efficacy Low immune response detected Infect both dividing and non-dividing cells	Limited cargo capacity (3.5–4 kb) High cost Technique expertise Safety obstacle Not easy to scale-up
Lentivirus	Expression stability Can be applied in a broad range of cell types Higher efficacy if constructs are shortened Low immune response detected Infect both dividing and non-dividing cells	Limited cargo capacity (8–9 kb) Arbitrary integration Technique expertise Safety obstacle Not easy to scale-up
Microinjection delivery is based on the use of a 0.5–5.0 µm diameter needle to deliver components into a cell or intercellular space, in this case the Cas9 protein and sgRNAs in any form. The electroporation method requires high voltage currents with the purpose of opening nanopores in the cellular membrane to inlet the CRISPR components resuspended in a specific buffer. Nucleofection is a specific electroporation-based method that allows the direct entry of the components into the nucleus. Lipofection consists in the introduction of the DNA components via a liposome-based transfection, in which synthetic cationic lipids aggregate around the negatively-charged DNA molecules. Cellular uptake is based on the fusion of these liposome-like structures with the phospholipidic membrane. Nanodiamonds are carbon nanomaterials which are suspended in a colloidal solution that allow the binding with or coating of biological material for cell transfection, mainly penetrating by the clathrin-mediated endocytosis pathway. The viral transduction method leverages the natural potential of viruses to infect cells, where the vectors have been deprived of the essential pathogenic genes in their replication. The most commonly used are AAVs and lentivirus. AAVs, which consist of single-stranded DNA, present several serotype versions (allowing tissue-specific transduction) and are considered a safe option, given that they are not associated to human diseases showing low immunogenicity and entailing low cellular toxicity (as they do not integrate into the host genome). Lentiviruses are retroviruses derived from a provirus of HIV that proffer stable expression in both dividing and post-mitotic cells due to their host genome integration, and that additionally accommodate cargos up to 5–6 kb in size.		
